# Stress accelerates hepatocellular carcinoma progression via a gut microbial-metabolite axis

**DOI:** 10.3389/fimmu.2026.1790214

**Published:** 2026-05-12

**Authors:** Zhe Hu, Pengfei Yue, Minlan Yuan, Zhenjie Zhang, Qiaoqi Li, Ting Yang, Yi Li, Bi-Sen Ding, Zhenxin Fan, Biao Yang, Zhongwei Cao

**Affiliations:** 1 Key Lab of Birth Defects and Related Diseases of Women and Children of MOE, State Key Lab of Biotherapy, State Key Laboratory of Respiratory Health and Multimorbidity, West China School of Basic Medical Sciences & Forensic Medicine, West China Second University Hospital, Sichuan University, Chengdu, China; 2 Abdominal Oncology Ward, Division of Medical Oncology, Cancer Center, West China Hospital, Sichuan University, Chengdu, Sichuan, China; 3 Mental Health Center, National Center for Mental Disorders, West China Hospital, Sichuan University, Chengdu, China; 4 Key Laboratory of Bioresources and Ecoenvironment (Ministry of Education), College of Life Sciences, Sichuan University, Chengdu, China; 5 West China Biomedical Big Data Center, West China Hospital, Sichuan University, Chengdu, China

**Keywords:** endothelial cells, gut microbiota, hepatocellular carcinoma, indole-3-propionic acid, stress, tumor microenvironment.

## Abstract

**Background:**

Hepatocellular carcinoma ranks among the most prevalent malignancies worldwide. While stress can modulate tumor initiation, progression, metastasis, and therapeutic response through diverse mechanisms, its specific role in hepatocellular carcinoma pathobiology remains elusive. This study aimed to elucidate the role of the gut microbiota in stress-promoted hepatocellular carcinoma progression and to uncover the pathways associated with disease progression.

**Methods:**

Integrating clinical and preclinical models, we delineated stress-induced restructuring of the gut microbiota and functionally restored specific microbial constituents. Mechanistic insights into the microbial metabolite indole-3-propionic acid were derived through *in vitro* and *in vivo* interrogations of the hepatocellular carcinoma tumor microenvironment.

**Results:**

Stress profoundly remodels the gut microbiota, with *Phocaeicola vulgatus* being significantly reduced. Restoration of *Phocaeicola vulgatus* or administration of its tryptophan-derived metabolite indole-3-propionic acid significantly attenuated hepatocellular carcinoma progression *in vivo*. Indole-3-propionic acid treatment reduced endothelial JAM2 expression and was associated with reduced JAM2–F11R-mediated endothelial–macrophage crosstalk in hepatocellular carcinoma, which may contribute to suppression of tumor progression.

**Conclusions:**

These findings support a role for the stress–gut microbiota–metabolite–tumor microenvironment axis in hepatocellular carcinoma progression and suggest potential translational targets for microbiome-based therapeutic strategies.

## 1 Introduction

Hepatocellular carcinoma (HCC) is the predominant histological subtype of primary liver cancer and ranks sixth in global incidence and third in cancer-related mortality (1). Well-characterized risk factors include chronic viral hepatitis, metabolic dysfunction-associated steatotic liver disease (MASLD), diabetes mellitus, and alcohol-related liver disease (2, 3). Although universal vaccination and potent antiviral therapies have reduced the burden of virus-driven HCC, the explosive increase in obesity and metabolic syndrome has rendered MASLD the most rapidly emerging etiology of HCC worldwide (4, 5).In addition, the gut microbiome has emerged as a critical modulator of hepatocarcinogenesis, influencing tumor initiation, progression, metastatic seeding, and therapeutic responsiveness (6–8). Notably, this microbial regulatory network does not exist in isolation, its functional homeostasis may be subject to multiple regulations by the systemic physiological state, among which stress has attracted increasing attention (9–11).

Major depressive disorder (MDD), one of the most prevalent psychiatric disorders worldwide and a leading cause of disability globally, exacerbates the global disease burden (12, 13). Patients with MDD exhibit gut microbiota dysbiosis, characterized by altered microbial diversity and functional disorders resulting from the disruption of intestinal ecological balance (14). The brain regulates this ecosystem via the autonomic nervous system by modulating intestinal peristalsis, secretion, permeability, and the release of hormones that regulate microbial gene expression, thereby reshaping the composition and activity of the gut microbiota (15–17). Cumulative evidence has shown that fecal microbiota analysis reveals MDD-related characteristic signatures: enrichment of Bacteroides, while a significant reduction in *Blautia*, *Faecalibacterium*, and *Coprococcus (*14, 18). Conversely, the microbiota communicates with the brain through neural, endocrine, and immune pathways: microbe-derived peptides and hormones enter the systemic circulation, activate vagal afferent nerves, spinal neurons, and the brain, thereby regulating gut-brain signaling (19–23). This bidirectional gut-brain axis regulatory network not only maintains the functional balance between the central nervous system and the gut, but its dysfunction may also affect the physiological and pathological states of distant organs through systemic regulation, which builds a crucial bridge for exploring the association between stress and liver diseases (15, 24).

As a core organ with direct anatomical and functional connections to the gut, the physiological and pathological processes of the liver are inevitably profoundly influenced by the homeostasis of the microbiota (25, 26). Previous studies have confirmed that gut microbiota dysbiosis or impaired barrier function is associated with liver diseases, suggesting that the pathogenesis of liver diseases is closely linked to gastrointestinal disorders (27, 28). Anatomically, venous blood from the small and large intestines drains into the portal vein, providing approximately 75% of hepatic blood flow; therefore, the liver is the first organ exposed to gut-derived nutrients and microbial metabolites (26, 29). Bidirectional microbiota-mediated crosstalk is further achieved through hepatic secretion of bile acids, IgA, and antimicrobial molecules into the small intestine, thereby completing a regulatory loop (25, 30). Metabolically, abundant commensal bacteria in the human gut—*Firmicutes*, *Bacteroidetes*, *Eubacterium*, and *Clostridium*—drive the conversion of primary bile acids to secondary bile acids (31). Imbalance in bile acid metabolism itself is also an important driving factor for the progression of liver diseases, further highlighting the central role of the gut-liver regulatory axis in the occurrence and development of liver diseases. Notably, homeostatic imbalance of this gut-liver regulatory axis, closely linked to liver disease progression, is likely to be intricately associated with HCC progression, laying a critical foundation for exploring crosstalk between gut microbes and HCC pathogenesis (32–34).

During the progression of HCC, the tumor microenvironment (TME), as the “intrinsic soil” for the survival and evolution of tumor cells (35), may be closely associated with the gut-liver regulatory axis. Its components include tumor cells, immune subsets, endothelial cells (ECs), fibroblasts, mesenchymal stem cells, and the extracellular matrix (ECM), cytokines, and chemokines secreted by them (36). Vascular endothelial cells line the luminal surface of blood vessels and lymphatics, forming a monolayer barrier; in addition to regulating gas and metabolite exchange, ECs play key regulatory roles in health and disease (37). Tumor-derived ECs are important stromal components of the TME and exert profound effects on tumor progression and metastasis (38, 39). However, the detailed mechanisms by which ECs shape the TME remain poorly understood, especially with respect to their association with the gut microecological regulatory network.

Collectively, although stress can remodel the gut microbiota via the gut–brain axis and gut microbiota dysbiosis is associated with myriad diseases (10, 40, 41), the role of gut microbiota in mediating the impact of stress-related mental illnesses on HCC and its underlying molecular mechanisms remain unclear. In this study, we integrated clinical and preclinical studies to dissect the host-microbe interactions underlying stress-accelerated HCC progression. We found that stress selectively reduces *P. vulgatus*, and is associated with decreased levels of the tryptophan-derived microbial metabolite indole-3-propionic acid (IPA). Loss of IPA was associated with altered intrahepatic endothelial cell–macrophage crosstalk and a shift toward an immunosuppressive tumor microenvironment, which may ultimately contribute to HCC progression.

## 2 Materials and methods

### 2.1 Major depressive disorder and healthy controls human subjects

A cohort study, approved by the Local Ethics Committee of West China Hospital, was performed. The publication of potentially identifying clinical information before enrollment (ClinicalTrials.gov NCT05010668). A written informed consent was obtained from each patient after being informed of the purpose and investigational nature of the present study. This study was conducted according to the Declaration of Helsinki, and strictly adhered to the CONSORT guidelines. From October 2021 to August 2023, 55 subjects diagnosed with MDD and 37 healthy individuals without a history of MDD were recruited from the Mental Health Center of West China Hospital, Sichuan University. The diagnosis was established using the Mini International Neuropsychiatric Interview (M.I.N.I.) (42), administered by senior psychiatrists.

Eligibility criteria for all participants in this study included: (1) aged between 18 and 65 years (inclusive); (2) educational attainment of primary school or higher, with sufficient comprehension of the study content; (3) provision of written informed consent after receiving a full explanation of the study aims and procedures; and (4) no psychotropic medication use for more than 2 weeks, or intermittent use for less than 3 days within a 2-week period.

Exclusion criteria included: (1) severe physical illness (such as malignant tumor, heart and respiratory failure), neurological disorders (such as cerebral infarction or neurodegenerative disorders); (2) schizophrenia, substance abuse, intellectual disability, anorexia nervosa/bulimia nervosa, or personality disorder as assessed by the M.I.N.I.; and (3) daily antibiotic or probiotic intake prior to enrollment.

### 2.2 Sample collection

Upon arrival at the Mental Health Center of West China Hospital, all participants completed a battery of psychological assessments, including the 17-item Hamilton Depression Rating Scale (HAMD-17) (43), Hamilton Anxiety Rating Scale (HAMA-14) (44), Patient Health Questionnaire (PHQ-9) (45), and 7-item Generalized Anxiety Disorder Scale (GAD-7) (46). These four scales were used collectively to evaluate the emotional status of participants. Demographic and clinical characteristics were also collected, including age, sex, smoking status, alcohol consumption, body mass index (BMI), religious affiliation, marital status, employment status, and monthly household income. Fresh stool and serum samples were collected from all participants on the day of enrollment. Additionally, each participant underwent a hepatic color Doppler ultrasound examination to confirm the presence of fatty liver disease and to rule out other hepatic disorders. The clinical samples utilized in this study partially overlap with those included in a prior investigation published by our research team (47).

### 2.3 Mice

All animal experiments were performed according to the guidelines approved by the Experimental Animal Ethics Committee of West China Hospital of Sichuan University (protocol code2020171A, date of approval 2 April 2019). SPF C57BL/6J mice (8 weeks of age, male) were obtained from GemPharmatech Co., Ltd. The mice were housed under a 12 h light/dark cycle, maintained with a regular chow diet, ambient temperature at 22-25°C and free access to water and food. Mice were randomized into groups before the beginning of treatment. For bacterial treatment, each mouse was given 200 μl of PBS daily by gavage containing 10^9^ CFU of bacteria starting one day post tumor cell engraftment until endpoint analysis. For metabolites treatment, each mouse was gavaged daily at 20 mg/kg body weight or 40 mg/kg body weight IPA or vehicle control starting one day post tumor cell engraftment until endpoint analysis.

### 2.4 Orthotopic HCC model

Orthotopic HCC was established in male C57BL/6J mice by a two-step protocol, as previously described (48). Step 1: Ascitic tumor propagation. H22 cells (1 × 10^7^ cells/mL in 0.2 mL sterile PBS) were injected intraperitoneally. Seven days later, 1–2 mL of hemorrhage-free ascites were harvested under aseptic conditions. Cervical dislocation was used for euthanasia on day 7 after ascites collection. Step 2: Orthotopic hepatic implantation. Ascitic fluid was pooled, diluted 1: 10 in ice-cold PBS, and centrifuged (300 g, 5 min, 4°C). After two additional washes, the pellet was resuspended in sterile 0.9% NaCl. Viability (> 95% by trypan-blue exclusion) was verified, and the suspension was adjusted to 2.5 × 10^7^ cells/mL and kept on ice. Mice were anaesthetized with 2% isoflurane in 100% O_2_, placed in supine position, and a 1 cm midline sub-xiphoid laparotomy was performed. The left lateral lobe was exteriorized and stabilized with sterile cotton swabs. Using a 30 G Hamilton syringe, 20 µL of the H22 suspension (5×10^5^ cells) was injected slowly at a 20° angle to a depth of 0.5 mm into the hepatic parenchyma. A steady and slow injection were performed and the needle was left *in situ* for 10 s to prevent leakage. The tract was compressed with sterile gauze until hemostasis was achieved. The abdominal wall and skin were closed with 5–0 Vicryl sutures. Animals recovered in a 37 ° C warming chamber and were returned to standard housing with ad libitum access to food and water. Cervical dislocation was used for euthanasia at indicated time points. Tumor volumes were calculated using the formula tumor volume= length x width^2^x 0.5, where length represents the largest tumor diameter and width represents the diameter perpendicular to the length.

### 2.5 Chronic unpredictable mild stress model

The CUMS treatment was performed according to published protocols (49, 50). Briefly, male C57BL/6J mice were exposed to CUMS beginning the day after the end of isolation. Mice in the CUMS groups (n=10) received two different stressors daily, with no individual stressor repeated on two consecutive days. Control mice were injected with the same tumor cells at the same time as the CUMS-exposed mice but were not subjected to any of the stressors. The CUMS protocol lasted for 21 consecutive days, behavioral testing started on day 22. Stressors were selected daily at random from the following list: tail pinch (5 minutes,1 cm from the distal portion of the tail); physical restraint, where mice were placed in a 50 mL plastic tube with both ends open to allow air for 60 minutes; sterile strange object exposure (24 hours); shaking the mouse cage (15 mins); odor irritation (3–4 hours); food deprivation (overnight); water deprivation (overnight); moist bedding (3–4 hours); removal of all bedding (3–4 hours); removal of all bedding and the addition of 30°C H2O (3–4 hours); 30° cage tilt (12 hours).

### 2.6 *P. vulgatus* and *Alistipes putredinis* (*A. putredinis*) administration

*P. vulgatus* and *A. putredinis* were obtained from ATCC. Both species were cultured in ATCC Medium 260 agar slant (ATCC)at 37°C under anaerobic condition. The bacterial cells of *P. vulgatus* and *A. putredinis* were pelleted by centrifugation at 10,000 rpm at 4°C for 10 min and then washed and re-suspended with sterile reduced PBS buffer to yield a suspension at a density of 10^9^ colony forming units (cfu). To get high-temperature inactivated *P. vulgatus* and *A. putredinis*, the bacteria were incubated at 95 °C for 150 minutes prior to gavage (51). The bacterial suspension was prepared freshly for animal experiments every day. *P. vulgatus* and *A. putredinis* culture were sequenced and then aligned to type strains with the Basic Local Alignment Search Tool (BLAST) to validate its purity before use.

### 2.7 Quantification and statistical analysis

All presented representative images were obtained from independently repeated experiments. Representative images from each individual group are presented in the figures. All data are presented as means ± SEM. Data was analyzed using an unpaired two-tailed Student’s t-test for single comparisons, and one-way or 2-way ANOVA for multiple comparisons. ANOVA analysis was followed by a Sidak’s *post-hoc* test. Survival data was analyzed by log-rank test. Correlations were calculated using the Spearman correlation. Figures and statistical analysis were generated using GraphPad Prism 9.5(GraphPad Software). The statistical test used, and P values are indicated in each figure legend. P values of < 0.05 were considered statistically significant. *P < 0.05, **P <0.01, ***P <0.001 and ****P <0.0001.

Additional materials and methods are provided in the **Supplementary Materials**.

## 3 Results

### 3.1 Stress accelerates HCC progression

**Figure 1:**
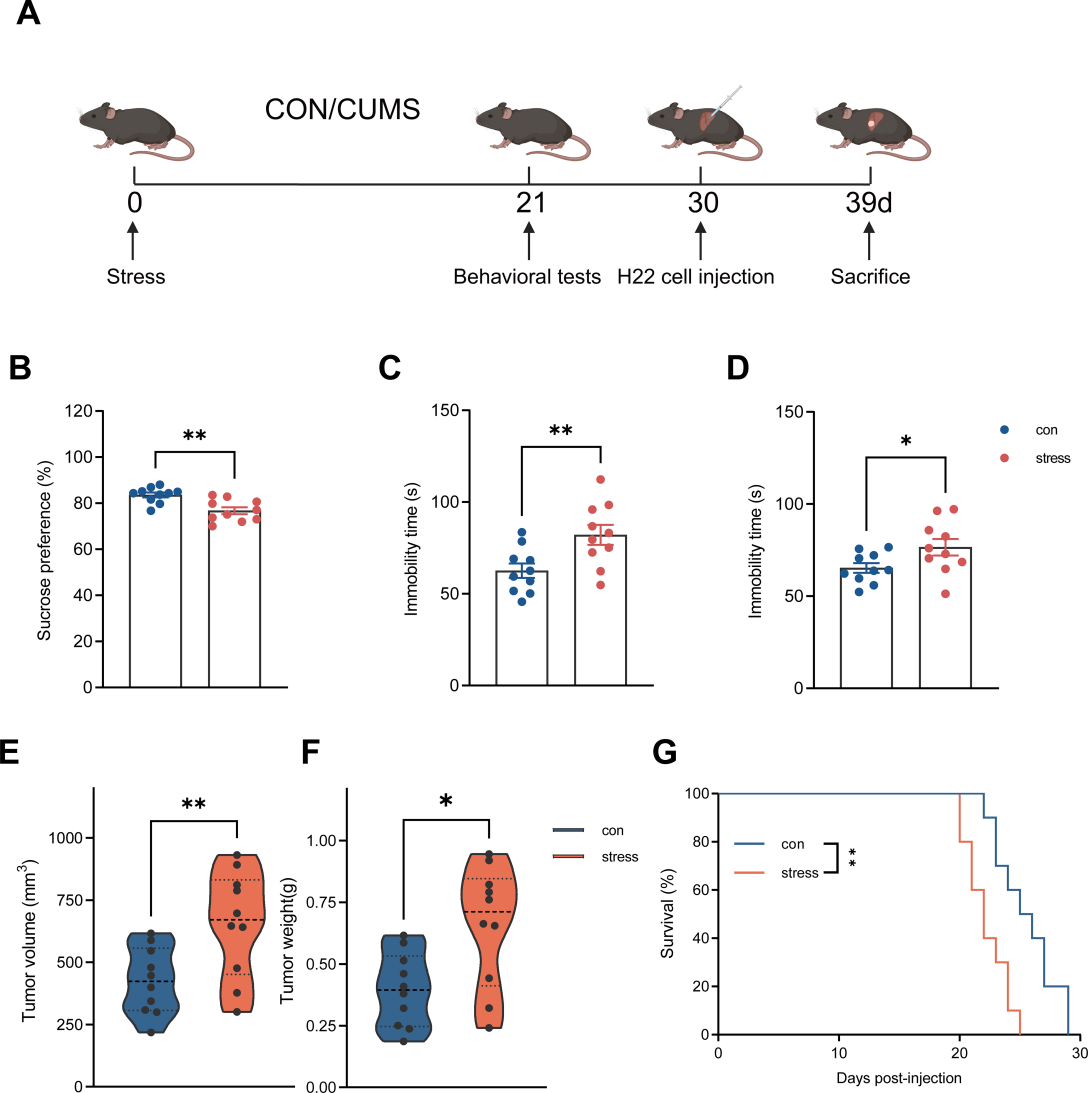
Stress accelerates HCC progression in preclinical model. **(A)** Schematic diagram showing the process of orthotopic HCC model after exposed to chronic restraint stress and their controls (n = 10 mice/group). **(B–D)** Behavioral test results for control and CUMS mice. Stress mice showed lower sucrose preference **(B)**, increased immobility time in the FST **(C)** and TST **(D)** (n = 10 mice/group). **(E–G)** Tumor volume, tumor weight and survival of h22 liver tumors from stress mice (stress) or control mice (con) (n = 10 mice/group). **(F)** Tumor volume. **(G)** Tumor weight. **(H)** Survival. **(B–D)** represent individual mice analyzed by unpaired t test. Mean ± SEM shown. **(E, F)** represent individual mice analyzed by unpaired t test, violin plot showing median and upper and lower quartiles. **(G)** represents survival curves analyzed by log-rank test. CUMS, unpredictable chronic mild stress. *p < 0.05, **p < 0.01.

Accumulating evidence supports a causal association between chronic stress and major depressive disorder, and several studies further suggest that stress-related psychiatric disorders accelerate the progression of HCC and other malignancies (52–56). To investigate the impact of chronic stress on tumor progression, we first applied the classical chronic unpredictable mild stress (CUMS) mouse model, a robust and translatable model for studying the neurobiological basis of MDD (49). Mice were subjected to two randomly selected stressors daily, and exhibited anxiety- and depression-like behavioral changes consistent with chronic-stress exposure (**Figure 1A**). In the sucrose-preference test, the stressed mice showed a reduced sucrose-preference ratio (**Figure 1B**), indicating anhedonia, which is the inability to experience pleasure from rewarding or enjoyable activities and is a core symptom of depression in humans (57). The stressed mice displayed a significantly prolonged immobility time in the forced-swim and tail-suspension tests (**Figures 1C, D**), indicating increased behavioral despair under CUMS conditions. After completion of the stress-model evaluation, mice were orthotopically transplanted with H22, a murine HCC cell line. Finally, stressed mice experienced a significantly accelerated progression of HCC and reduced survival (**Figures 1E–G**). Together, these results indicate that chronic stress promoted HCC progression in mice.

**Figure 2:**
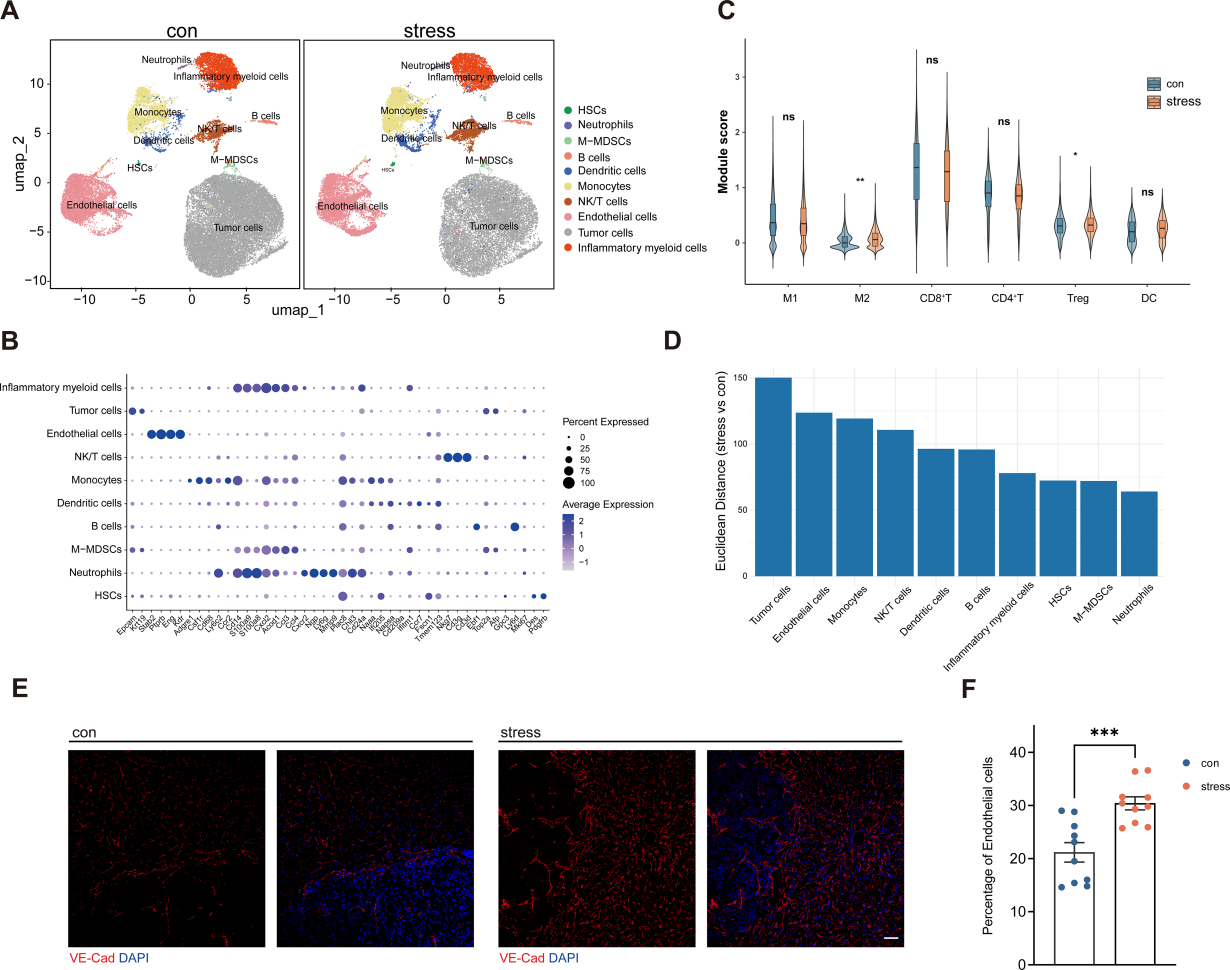
Stress remodels the HCC tumor microenvironment. **(A)** Single-cell RNA sequencing and uniform manifold approximation and projection (UMAP) clustering of tumor tissue cells from H22 tumor-bearing mice that had previously undergone stress or control modeling (n = 3 mice/group). **(B)** Dot plot showing expression of marker genes for identification of cluster cell types of tumor tissue. Dot size is proportional to the percentage of each cluster expressing the marker gene, and the color intensity is correlated with the expression level. **(C)** Module score analysis of major immune populations in mouse HCC samples from control and stress groups (n = 3 mice/group). **(D)** Euclidean distance analysis of the similarity of gene expression profiles between stress or control group in pseudobulk analysis. **(E)** Immunostaining of VE-cadherin (red) on tumor sections from control and stress mice. **(F)** Percentage of endothelial cells in h22 tumor tissues of control and stress mice, assessed by flow cytometry (n = 10 mice/group). **(F)** represents individual mice analyzed by unpaired t test. Mean ± SEM shown. HSCs, hepatic stellate cells; M-MDSCs, monocytic myeloid-derived suppressor cells; NK/T cells, natural killer/T cells (NK/T cells). ns, not significant, *p < 0.05, **p < 0.01, ***P <0.001.

To investigate the impact of chronic stress on HCC progression in mice, we performed single-cell RNA-sequencing (scRNA-seq) analysis of tumor tissues of either stressed mice or control mice. Unsupervised clustering identified 10 distinct cell clusters, including tumor cells, ECs, hepatic stellate cells (HSCs), neutrophils, monocytic myeloid-derived suppressor cells (M-MDSCs), B cells, dendritic cells (DCs), monocytes, natural killer/T cells (NK/T cells), and inflammatory myeloid cells (**Figures 2A, B**). To further characterize stress-associated immune landscape changes in the TME, we performed immune cell signature scoring based on the scRNA-seq dataset. The analysis showed significant enrichment of Treg cells and M2-like macrophages in stressed mice, suggesting a stress-associated shift toward an immunosuppressive tumor microenvironment. In contrast, CD8^+^ T cells, CD4^+^ T cells, dendritic cells (DCs), and M1-like macrophages did not show significant differences between stressed and control mice in the current dataset (**Figure 2C**). Tumor vascular ECs are key functional regulators of the HCC tumor microenvironment (58, 59). Relative to control mice, pseudobulk transcriptional analysis further demonstrated that stress exposure markedly remodeled the EC transcriptomic profile in stress mice (**Figure 2D**). Similarly, immunofluorescent staining and flow cytometry analysis revealed an increased proportion of ECs in tumors from stressed mice (**Figures 2E, F**). Taken together, these findings suggest that stress accelerates HCC progression in association with EC remodeling in the TME and a stress-associated shift toward an immunosuppressive tumor microenvironment, particularly reflected by increased Treg enrichment and M2-like macrophage abundance.

### 3.2 Stress promotes HCC by altering the gut microbiota micro-environment

**Figure 3:**
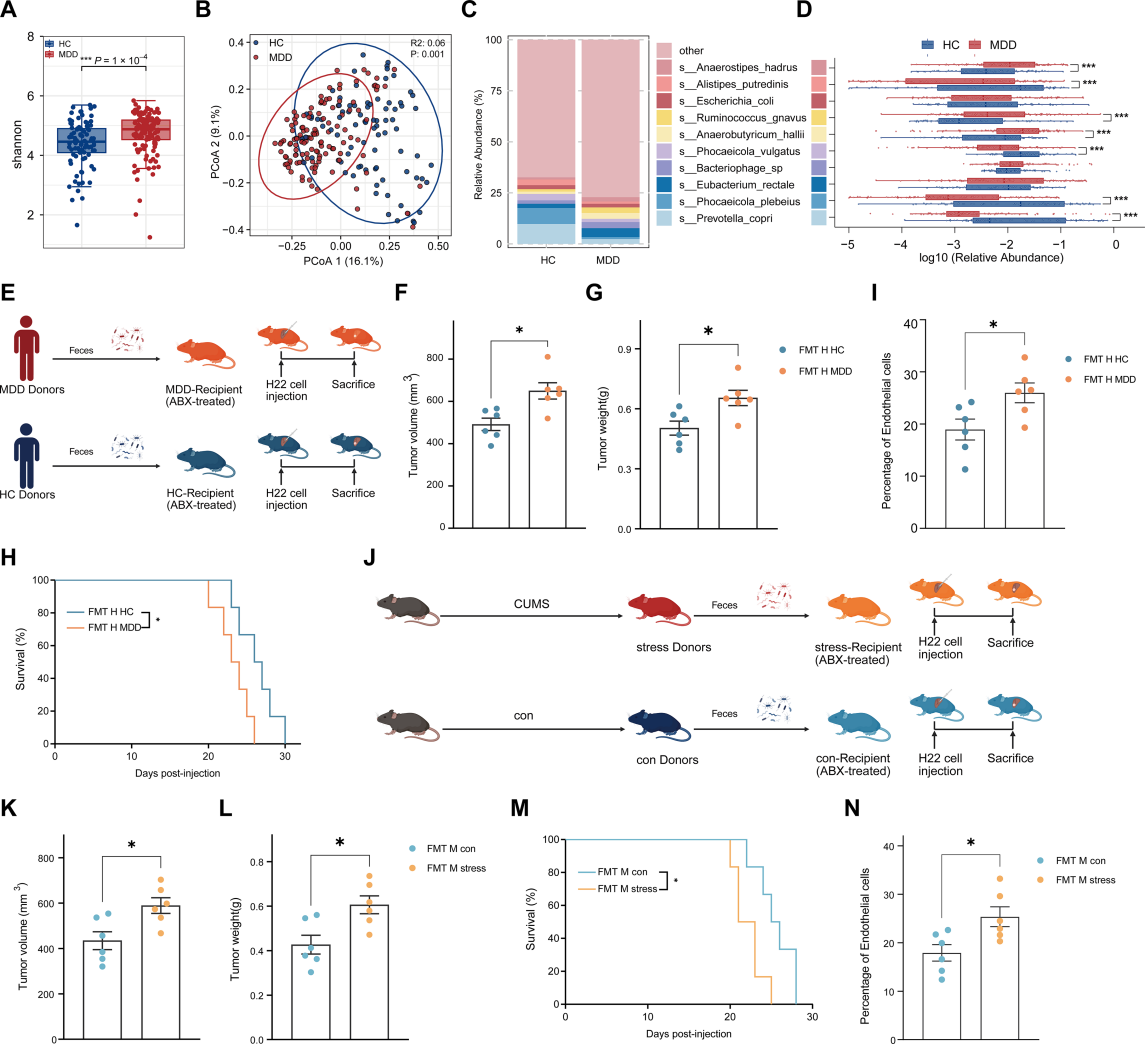
Stress-induced gut microbiota dysbiosis promotes HCC progression in human and mice models. **(A)** Alpha diversity indices (Shannon) at the species level, comparing microbial richness and evenness between MDD and HC human subjects. **(B)** Principal coordinates analysis (PCoA) of species-level microbial composition between MDD and HC human subjects. **(C)** Average relative abundance of the top 10 bacterial species in MDD and HC human subjects. **(D)** Box plots with overlaid dot plots showing the log10 relative abundance of top 10 bacterial species that were differentially abundant in HC and MDD human subjects. **(E)** Schematic diagram showing the human fecal microbiota transplantation process. Fresh stool samples were prepared and transferred to recipient mice before orthotopic HCC model. **(F–H)** Tumor volume, tumor weight and survival of h22 tumors of FMT H MDD or FMT H HC mice (n = 6 mice/group). **(F)** Tumor volume. **(G)** Tumor weight. **(H)** Survival. **(I)** Percentage of endothelial cells in h22 tumor tissues of FMT H MDD or FMT H HC mice, assessed by flow cytometry (n = 6 mice/group). **(J)** Schematic diagram showing the mice fecal microbiota transplantation process. Fresh stool samples from stress or con mice were prepared and transferred to recipient mice before orthotopic HCC model. **(K–M)** Tumor volume, tumor weight and survival of h22 tumors of FMT M stress or FMT M con mice (n = 6 mice/group). **(K)** Tumor volume. **(L)** Tumor weight. **(M)** Survival. **(N)** Percentage of endothelial cells in h22 tumor tissues of FMT M stress or FMT M con mice, assessed by flow cytometry (n = 6 mice/group). **(F, G, I, K, L, N)** represent individual mice analyzed by unpaired t test. Mean ± SEM shown. **(H, M)** represent survival curves analyzed by log-rank test. ABX-treated, antibiotic-treated; FMT H HC, fecal microbiota transplantation human HC; FMT H MDD, fecal microbiota transplantation human MDD; FMT M con, fecal microbiota transplantation mice con; FMT M stress, fecal microbiota transplantation mice stress. *p < 0.05, ***p < 0.001.

Recently, increasing evidence has indicated that gut microbiota dysbiosis might be implicated in the physiological mechanisms of neuropsychiatric disorders. Altered microbial community composition, diversity and distribution traits have been reported in neuropsychiatric disorders (60, 61). Thus, to explore whether the tumor-promoting effect of stress on liver cancer is related to alterations in the gut microecosystem, we first collected fecal samples from 55 patients with MDD and from 37 healthy controls (HC) for metagenomic sequencing. The MDD group exhibited a significant increase in alpha diversity, indicating increased microbial richness under MDD (**Figure 3A**). Principal coordinates analysis (PCoA) revealed a significant difference in microbial community structure between the HC and MDD groups, indicating that stress can induce large-scale alterations in the gut microbiota in different directions (**Figure 3B**). Microbiome profiling at the species level revealed a distinct composition between the HC and MDD groups, especially with *P. vulgatus*, *A. putredinis*, *Phocaeicola plebeius* and *Prevotella copri* significantly enriched in the HC group, whereas *Anaerostipes hadrus*, *Ruminococcus gnavus* and *Anaerobutyricum hallii* dominated the MDD cohort (**Figures 3C, D**). Collectively, these results identify stress-associated alterations in the gut microecosystem in the MDD cohort.

To further determine whether stress influences HCC progression via the gut microbiota, we gavage-transplanted fecal samples from 20 depressed patients and 12 healthy controls into antibiotic-pretreated SPF recipient mice (**Figure 3E**). Mice (MDD-Recipient) receiving feces from depressed patients (MDD-Donors) exhibited significantly accelerated liver-cancer progression, reduced survival and a substantially elevated proportion of tumor ECs within the HCC TME compared with those mice (HC-Recipient) receiving feces from healthy controls (HC- Donors) (**Figures 3F–I**). Additionally, a similar trend was observed in antibiotic-pretreated mice (stress-Recipient or con-Recipient as indicated) following fecal transplantation from stress or control mice (**Figures 3J–N**). These data support a causal role for stress-associated gut microbiota in promoting HCC development in mouse models.

### 3.3 *P. vulgatus* can inhibit liver-cancer progression

**Figure 4:**
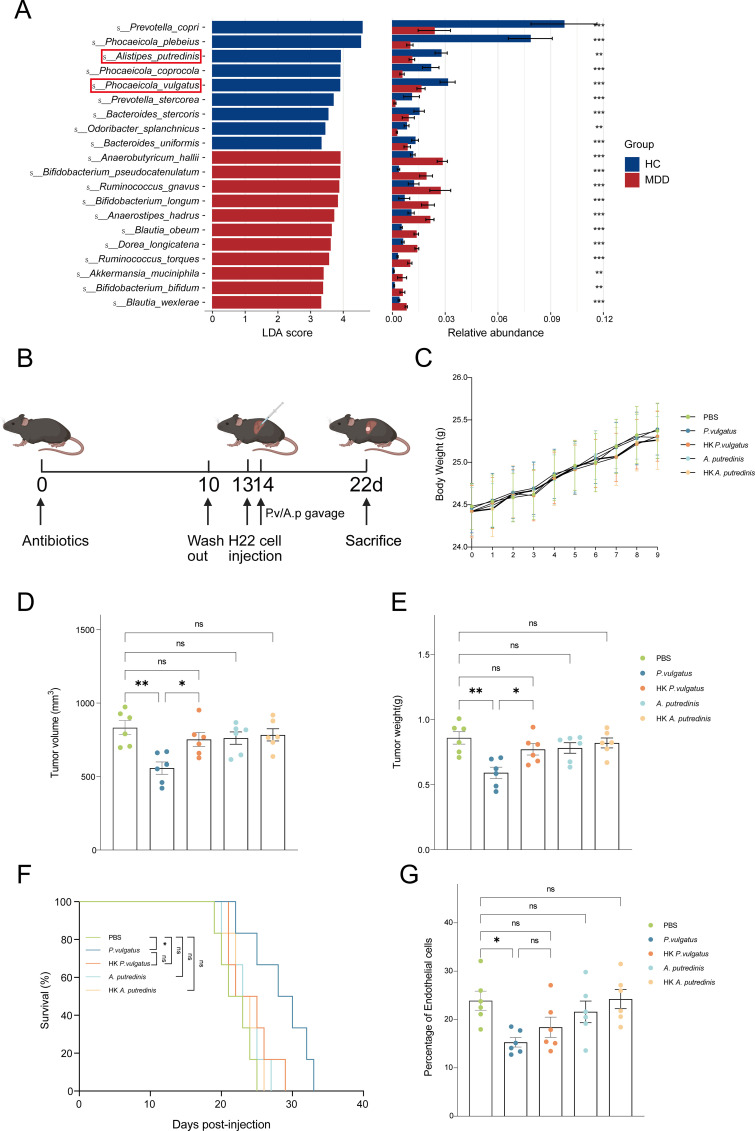
P*. vulgatus* suppresses HCC progression via its metabolic activity. **(A)** LEfSe analysis showing the top 20 discriminative taxa ranked by LDA score in MDD and HC human subjects (LDA score > 2, P < 0.05). **(B)** Schematic diagram showing Abx pretreated h22 tumor-bearing mice treated with PBS, *P. vulgatus*, HK *P. vulgatus*, *A. putredinisp* or HK *A. putredinisp*. **(C)** Body weight changes in mice treated with PBS, *P. vulgatus*, HK *P. vulgatus*, *A. putredinisp* or HK *A. putredinisp* during the tumor-bearing period (n = 6 mice/group). **(D–F)** Tumor volume, tumor weight and survival of h22 tumor-bearing mice treated with PBS, *P. vulgatus*, HK *P. vulgatus*, *A. putredinisp* or HK *A. putredinisp* treated mice (n = 6 mice/group). **(C)** Tumor volume. **(D)** Tumor weight. **(E)** Survival. **(G)** Percentage of endothelial cells in h22 tumor-bearing mice treated with PBS, *P. vulgatus*, HK *P. vulgatus*, *A. putredinisp* or HK *A. putredinisp*, assessed by flow cytometry (n = 6 mice/group). **(C–E, G)** represent individual mice analyzed by one-way ANOVA with Sidak’s correction for multiple comparisons. Mean ± SEM shown. **(F)** represent survival curves analyzed by log-rank test. *P. vulgatus*, live *P. vulgatus*; HK *P. vulgatus*, heat-killed *P. vulgatus*; *A. putredinisp*, live *A. putredinisp*; HK *A. putredinisp*, heat-killed *A. putredinisp*. ns, not significant, *p < 0.05, **p < 0.01.

To further identify potential bacteria that play an important role in the process of stress-promoting HCC progression, we analyzed the top 20 bacterial species with the most significant differential abundance in fecal metagenomic sequencing data between the MDD group and the HC group, both *P. vulgatus* and *A. putredinis* were significantly down-regulated in MDD patients and exhibited higher relative abundance (**Figure 4A**). Thus, we further proceeded to investigate *in vivo* whether and how *P. vulgatus* and *A. putredinis* modulate HCC progression. We administered mice with heat-killed *P. vulgatus* (HK *P. vulgatus*), heat-killed *A. putredinis* (HK *A. putredinis*), live *P. vulgatus* (*P. vulgatus*), or live *A. putredinis* (*A. putredinis*) (**Figure 4B**). Mouse body weight was monitored throughout the treatment period, and no abnormal weight loss was observed under the experimental conditions used in this study (**Figure 4C**). Only live *P. vulgatus* significantly suppressed HCC progression, extended survival and reduced proportion of tumor ECs (**Figures 4D–G**), indicating that the physical components of *P. vulgatus* do not exert anti-tumor effects. These findings indicate that the anti-tumor effect of *P. vulgatus* is mediated by its metabolic activity rather than by its physical components alone. Taken together, *P. vulgatus*, which was significantly downregulated in the MDD group, exerts a suppressive effect on HCC progression.

### 3.4 Tryptophan-derived metabolite IPA markedly suppresses HCC progression

**Figure 5:**
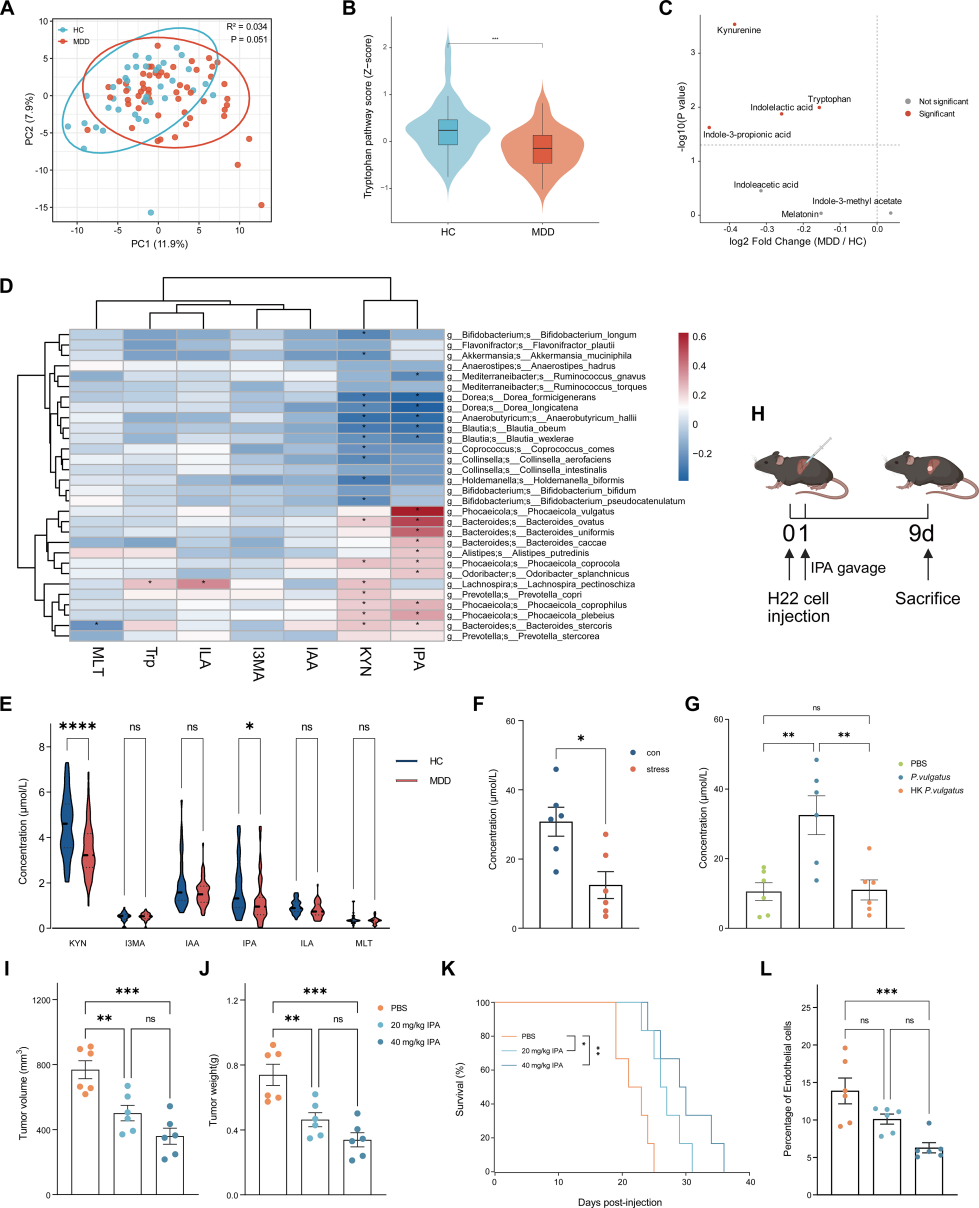
Microbiota-derived IPA suppresses HCC progression. **(A)** Principal component analysis (PCA) of serum metabolites in HC and MDD human subjects. **(B)** Tryptophan pathway analysis of differentially enriched serum metabolites in HC and MDD human subjects. **(C)** Volcano plots of differential tryptophan-related serum metabolites in HC and MDD human subjects. **(D)** Spearman correlation heatmap between top 30 differential microbial species and tryptophan-related serum metabolites. **(E)** Concentrations of tryptophan-related serum metabolites in HC and MDD human subjects. **(F)** IPA concentrations in control or stress mice (n = 6 mice/group). **(G)** IPA concentrations in mice orally gavaged with PBS, *P. vulgatus* or HK *P. vulgatus* (n = 6 mice/group). **(H)** Schematic diagram showing h22 tumor-bearing mice orally administered IPA (20 mg/kg body weight [b.w.] or 40 mg/kg [b.w.]) or vehicle control (PBS) as indicated. **(I–K)** Tumor volume, tumor weight and survival of h22 tumor-bearing mice treated with IPA (20 mg/kg body weight [b.w.] or 40 mg/kg [b.w.]) or vehicle control (PBS) (n = 6 mice/group). **(I)** Tumor volume. **(J)** Tumor weight. **(K)** Survival. **(L)** Percentage of endothelial cells in h22 tumor-bearing mice treated with IPA (20 mg/kg body weight [b.w.] or 40 mg/kg [b.w.]) or vehicle control (PBS), assessed by flow cytometry (n = 6 mice/group). **(E)** represent individual human subjects analyzed by two-way ANOVA with Sidak’s correction for multiple comparisons. Mean ± SEM shown. Violin plot showing median and upper and lower quartiles. **(F)** represent individual mice analyzed by unpaired t test. Mean ± SEM shown. **(G)**, **(I, J)** and **(L)** represent individual mice analyzed by one-way ANOVA with Sidak’s correction for multiple comparisons. Mean ± SEM shown. **(K)** represent survival curves analyzed by log-rank test. KYN, kynurenine; I3MA, indole-3-methyl acetate; IAA, indole-3-acetic acid; IPA, indole-3-propionic acid; ILA, indole-3-lactic acid; MLT, melatonin. ns, not significant, *p < 0.05, **p < 0.01, ***p < 0.001, ****p < 0.0001.

Serum metabolomic profiling revealed a significant difference in metabolite composition between MDD and HC groups (**Figure 5A**), and the HC group showed significant enrichment of the tryptophan metabolism pathway (**Figure 5B**). Tryptophan is an essential amino acid in humans and can be converted by microbial indole pathways into indole derivatives such as IPA and indole-3-lactic acid (ILA) (62, 63). Accordingly, among the tryptophan-derived indole metabolites downregulated in the MDD group, IPA was significantly decreased (**Figure 5C**). IPA is an indole analog specifically produced by the gut microbiota and has been shown to promote axonal regeneration and to enhance immune-checkpoint blockade efficacy in multiple cancers (64). Spearman correlation analysis demonstrated a significant correlation between *P. vulgatus* and IPA (**Figure 5D**). Consistent with the above findings, both MDD group and stressed mice displayed lower plasma IPA levels (**Figures 5E, F**). Moreover, oral gavage with *P. vulgatus* significantly increased serum IPA levels *in vivo* (**Figure 5G**). We therefore verified the potential impact of IPA on HCC progression *in vivo*. Intragastric administration of IPA to mice significantly inhibited HCC progression in a dose-dependent manner (**Figures 5H–K**). IPA supplementation markedly reduced the proportion of ECs within tumor tissues (**Figure 5L**). Taken together, these findings support a tumor-suppressive role for IPA in HCC progression.

### 3.5 IPA is associated with reduced JAM2–F11R-mediated endothelial–macrophage crosstalk in HCC

**Figure 6:**
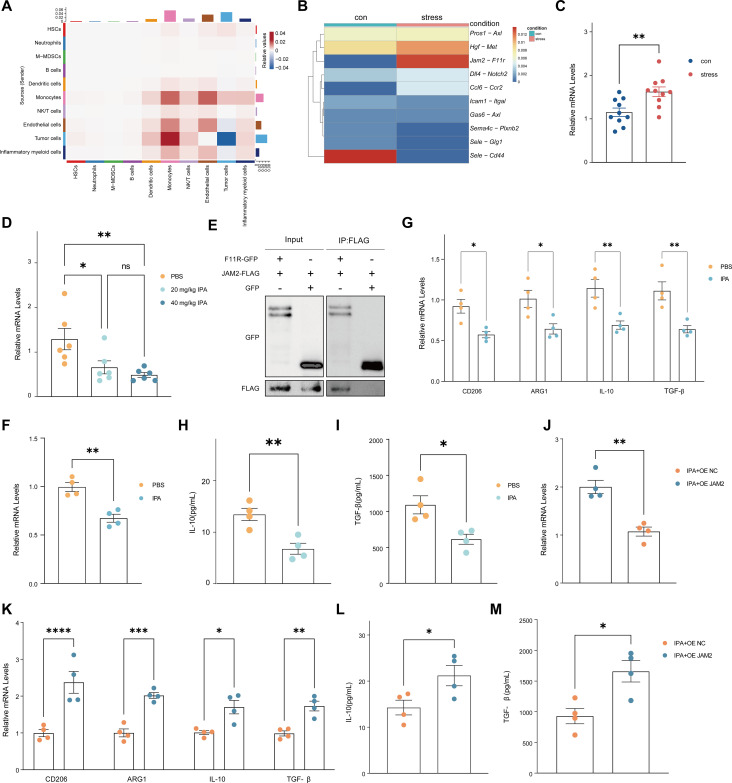
IPA is associated with reduced JAM2–F11R-mediated endothelial–macrophage crosstalk in HCC. **(A)** Heatmap of cell-cell interaction strength in HCC by analysis of ligand-receptor interactions between distinct cell types were shown. **(B)** Heatmap of top ten differential ligand-receptor interactions between macrophages and endothelial cells. **(C)** Relative mRNA levels of Jam2 in endothelial cells of HCC tumors from control and stress mice, measured by RT-qPCR (n = 6/group). **(D)** Relative mRNA levels of Jam2 in endothelial cells of HCC tumors from mice treated with IPA (20 mg/kg body weight [b.w.] or 40 mg/kg b.w.) or vehicle control (PBS), measured by RT-qPCR (n = 6/group). **(E)** Co-IP demonstrated the protein interaction between JAM2 and F11R in 293T cells. **(F)** Relative mRNA levels of JAM2 in HUVECs treated with IPA or PBS, measured by RT-qPCR. **(G)** Relative mRNA levels of the M2 macrophage markers CD206, arginase-1 (Arg1), interleukin 10 (IL-10) and transforming growth factor-β (TGF -β) in the upper THP-1 cell chambers in the macrophage-endothelial co-culture. HUVECs were pretreated with PBS or IPA. **(H, I)** ELISA analysis of supernatant for IL-10 **(H)** and TGF-β1 **(I)** from THP-1 macrophages in the upper chambers in the macrophage-endothelial co-culture. HUVECs were treated with PBS or IPA. **(J)** Overexpression of JAM2 in HUVECs modulating THP-1 macrophage M2 polarization in the endothelial-macrophage co-culture. HUVECs were transduced with lentivirus overexpressing scrambled sequence as negative control (OE-NC) or JAM2 (OE-JAM2). **(K)** Relative mRNA levels of the M2 macrophage markers CD206, Arg1, IL-10 and TGF-β1 in THP-1 cells, measured by RT-qPCR. HUVECs were pre-transduced with lentivirus overexpressing scrambled sequence as negative control (OE-NC) or JAM2 (OE-JAM2). **(L, M)** ELISA analysis of supernatant for IL-10 **(L)** and TGF-β1 **(M)** from THP-1 macrophages in the upper chambers in the macrophage-endothelial co-culture. HUVECs were pre-transduced with lentivirus OE-NC or OE-JAM2. **(C, F, H, I, J, L, M)** represents 4 independent samples per group analyzed by unpaired t test. Mean ± SEM shown. **(D)** represents individual mice analyzed by one-way ANOVA with Sidak’s correction for multiple comparisons. Mean ± SEM shown. **(G, K)** represents 4 independent samples per group analyzed by two-way ANOVA with Sidak’s correction for multiple comparisons. Mean ± SEM shown. HSCs, hepatic stellate cells; M-MDSCs, monocytic myeloid-derived suppressor cells; NK/T cells, natural killer/T cells (NK/T cells); IPA, indole-3-propionic acid. ns, not significant, *p < 0.05, **p < 0.01, ***p < 0.001, ****p < 0.0001.

Tumor vascular ECs are stromal cells that play a fundamental role in the TME. However, the precise molecular mechanism by which the gut-microbe-derived metabolite IPA regulates endothelial signals during HCC progression remains incompletely understood. To clarify how IPA influences HCC progression through vascular ECs, we performed analysis of scRNA-seq data from tumor tissues of stressed versus control mice. Cell-interaction analysis found that the interaction strength between ECs and macrophages was significantly enhanced (**Figure 6A**) and that the interactions between vascular ECs and macrophages via the JAM2–F11R axis were significantly enhanced in stress mice (**Figure 6B**). Consistently, endothelial cells isolated from stressed mice exhibited significantly increased Jam2 mRNA expression compared with controls (**Figure 6C**). RT–qPCR profiling of endothelial cells isolated from IPA-treated HCC-bearing mice revealed that only Jam2 was significantly down-regulated following IPA administration among the genes examined (**Figure 6D**; **Supplementary Figure 1A**). Furthermore, co-immunoprecipitation (co-IP) confirmed a direct interaction between JAM2 and F11R (**Figure 6E**). JAM2 is predominantly expressed in ECs, participates in regulating tight junctions, and is involved in leukocyte migration and inflammatory responses, thereby playing an important role in the immune system (65). Treatment of cultured human umbilical vein ECs (HUVECs) with IPA significantly down-regulated JAM2 expression (**Figure 6F**). Next, we co-cultured differentiated THP-1 monocytes with IPA-treated HUVECs. Relative to PBS-treated controls, THP-1 cells exhibited a marked reduction of M2-polarization markers (66), including CD206, ARG1 (Arginase 1), IL-10 (Interleukin 10) and TGF-β (Transforming Growth Factor-β), whereas M1-polarization markers exhibited no significant alterations (**Figure 6G**; **Supplementary Figure 1B**). Direct exposure of differentiated THP-1 cells to IPA alone was insufficient to induce M2 polarization, indicating that ECs are required for IPA-dependent modulation of macrophage phenotype (**Figure 6G**; **Supplementary Figure 1C**). ELISA assays confirmed significant down-regulation of the M2 cytokines IL-10 and TGF-β (**Figures 6H, I**). However, when JAM2-overexpressing ECs were co-cultured with differentiated THP-1 cells, IPA failed to elicit the above changes (**Figures 6J–M**), suggesting that the JAM2–F11R axis contributes to endothelial–macrophage crosstalk and macrophage polarization. In summary, IPA was associated with reduced JAM2–F11R-mediated endothelial–macrophage interactions in HCC, which may contribute to suppression of tumor progression.

## 4 Discussion

Over the past two decades, converging evidence has established that the gut microbiota exerts a profound capacity to modulate brain activity and behavior; conversely, signals originating from the brain can reshape microbial composition and function (67, 68). Microbe–host communication proceeds via immune, neuroendocrine, and neuronal routes that collectively constitute the brain–gut–microbiota axis (69). Pre-clinical studies demonstrate that microorganisms exploit this bidirectional network to influence neurodevelopment, synaptic function, and behavioral outputs (70). Clinically, initial surveys reveal altered fecal bacterial profiles in patients with major depressive disorder relative to healthy controls (71). In our study, the MDD cohort was used to identify stress-associated microbial signatures, whereas fecal microbiota transplantation into antibiotic-pretreated specific-pathogen-free mice and mechanistic studies in mouse models supported a causative role of these microbiota in promoting HCC progression. Nevertheless, antibiotic treatment does not achieve absolute sterility; validation in germ-free animals is therefore warranted to definitively attribute the observed phenotype to microbial absence.

*P. vulgatus*—formerly classified within Bacteroides—is a Gram-negative commensal specialized in the fermentation of complex host glycans such as glycogen, thereby generating energy and essential nutrients (72, 73). Several reports have attributed beneficial effects to this organism, and recent work identified *P. vulgatus* as a candidate probiotic for MASLD (74). Mechanistically, *P. vulgatus*-derived 3-hydroxyphenylacetic acid (3-HPAA) attenuates hepatic steatosis by reducing histone H3 acetylation and suppressing squalene epoxidase (SQLE) expression—an enzyme critical for cholesterol biosynthesis. *Phocaeicola* also have been associated with alleviation of atherosclerosis and colitis, although the underlying molecular mechanism remains unclear (75, 76). Conversely, positive associations between *P. vulgatus* and Crohn’s disease or type 2 diabetes have also been reported, underscoring its context-dependent dualism (77, 78). The microbial community is a dynamic network whose composition fluctuates with diet, lifestyle, and disease state, complicating univariate interpretations. In the present study, our data support the hypothesis that *P. vulgatus* restrains HCC progression, at least in part, in association with restoration of circulating IPA; however, the genomic determinants enabling *P. vulgatus* to channel tryptophan into IPA remain unresolved. During bacterial gavage, no overt body weight changes were observed, although food and water intake were not systematically recorded; more comprehensive safety evaluation will be important in future studies. Metagenomic mining reveals candidate tryptophanase and decarboxylase loci, yet whether *P. vulgatus* produces IPA autonomously or via cross-feeding interactions awaits functional validation.

A substantial fraction of dietary tryptophan is converted by microbial tryptophanases and decarboxylases into bioactive indole derivatives—including indole-3-lactate (ILA), indole-3-propionate (IPA), indole-3-acetate (IAA), and indole-3-acetamide (IAM)—that engage host aryl-hydrocarbon receptor (AhR) and pregnane X receptor (PXR) pathways (79, 80). AhR and PXR are common target receptors for indole metabolites *in vivo*; current evidence shows that AhR and PXR in intestinal epithelium can exert dual roles: these receptors not only protect the intestinal epithelium from inflammatory damage and contribute to maintaining epithelial barrier integrity, but also participate in regulating the behavior of transformed colonic cells, posing a potential risk of promoting cancer progression (81–84). Studies have indicated that IPA can effectively prevent chronic colonic inflammation by activating AHR (85). However, in our study, stress was associated with increased endothelial JAM2 expression *in vivo*, whereas IPA treatment reduced JAM2 expression. However, the precise mechanism by which IPA regulates JAM2 transcription remains unclear and requires further investigation. In addition, whether other indole metabolites influence HCC progression also warrants further study.

The TME is a hierarchically organized ecosystem composed of malignant cells, innate and adaptive immune subsets, ECs, cancer-associated fibroblasts, soluble mediators (cytokines, chemokines, growth factors), and a remodeled extracellular matrix (86). Once considered passive bystanders, TME constituents are now recognized as active drivers of tumorigenesis and attractive therapeutic targets (87). Hepatic ECs not only regulate vascular tone and perfusion but also orchestrate immune trafficking through secretion of angiocrine and immunomodulatory cues, thereby directly modulating tumor-cell behavior and anti-tumor immunity (88). Our data implicate the JAM2–F11R axis in regulation of the HCC immune microenvironment. In line with the scRNA-seq findings, stress was associated with a tendency toward an immunosuppressive microenvironment, particularly at the level of Treg enrichment and macrophage polarization, while additional validation of other immune populations will be valuable in future studies. The direct impact of *in vivo* JAM2–F11R blockade on tumor progression remains to be further validated.

## Data Availability

The raw metagenomic sequencing data have been deposited in the National Genomics Data Center (NGDC) (https://ngdc.cncb.ac.cn/) under accession number PRJCA043176. The processed single-cell RNA-seq data and associated metadata are available under GEO Series accession number GSE330363.
