# Supplementary material for: Stress accelerates hepatocellular carcinoma progression via a gut microbial-metabolite axis
Source: Front Immunol. 2026 May 12;17:1790214. doi: 10.3389/fimmu.2026.1790214 (PMC13205688; doi:10.3389/fimmu.2026.1790214)
Supplement: Supplementary file 1 [file DataSheet1.docx]

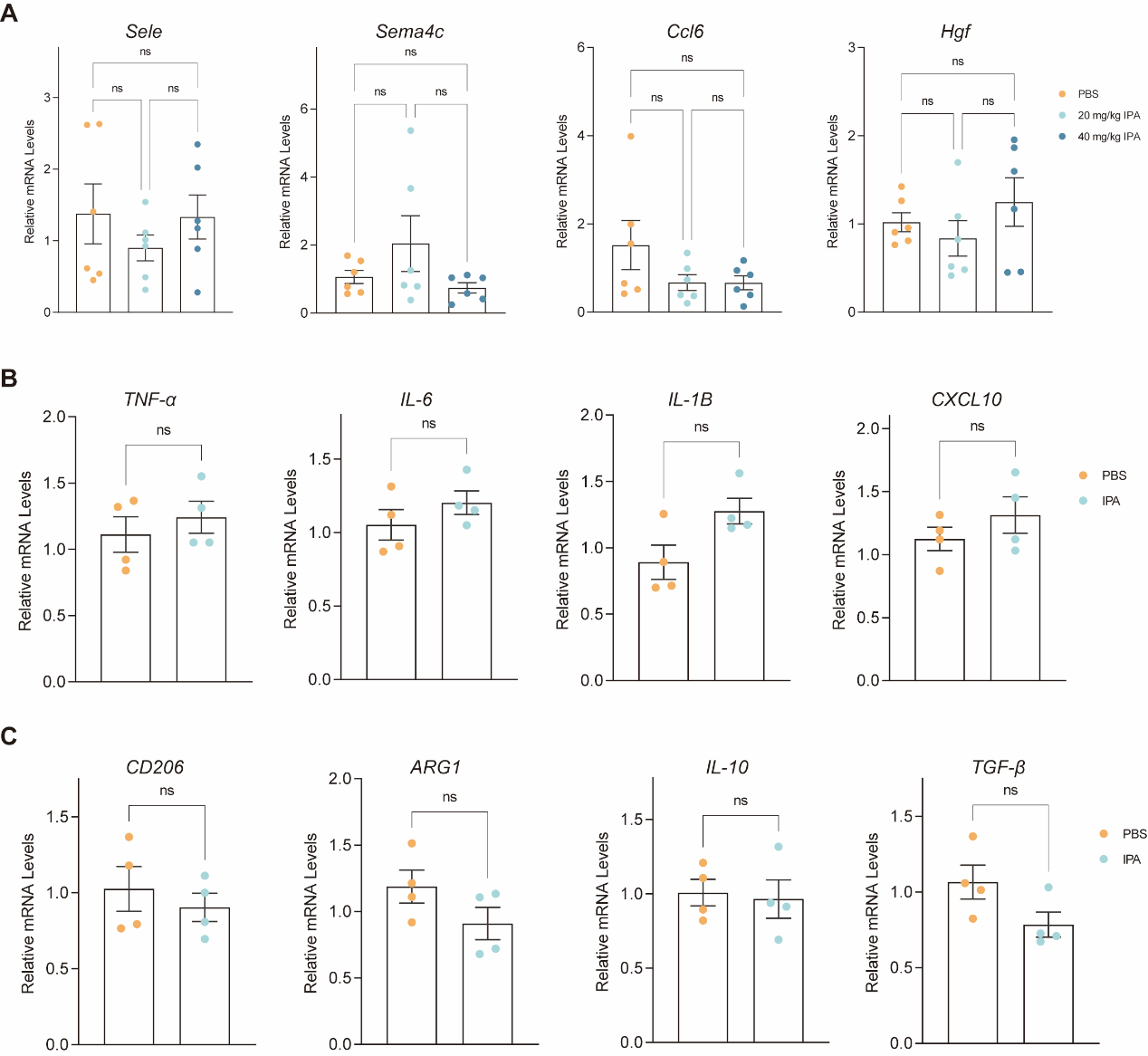


**Supplementary Figure 1. Validation of IPA-mediated regulation of JAM2 expression and macrophage polarization via endothelial cells**

(A) Relative mRNA levels of *Sele*, *Sema4c*, *Ccl6* and *Hgf* in endothelial cells of HCC tumors from mice treated with IPA (20 mg/kg body weight [b.w.] or 40 mg/kg b.w.) or vehicle control (PBS), measured by RT-qPCR (n = 6/group).

(B) Relative mRNA levels of the M1 macrophage markers tumor necrosis factor-α (TNF-α), interleukin-6 (IL-6)， interleukin-1B (IL-1B) and C-X-C motif chemokine ligand 10 (CXCL10) in the upper THP-1 cell chambers in the macrophage-endothelial co-culture. HUVECs were pretreated with PBS or IPA.

(C) Relative mRNA levels of the M2 macrophage markers CD206, Arg1, IL-10 and TGF -β in THP-1 macrophages treated with IPA or PBS, measured by RT-qPCR.

(A), (B) and (C) represents 4 independent samples per group analyzed by unpaired t test. Mean ± SEM shown. ns, not significant.

**Supplementary Table 1. List of Bacteria specific 16S rRNA PCR primers.**

| Code | Sequence 5'–3' |
| --- | --- |
| 16S 27F | GTTTGATCCTGGCTCAG |
| 16S 1492R | CGGCTACCTTGTTACGAC |
| 16S 338F | ACTCCTACGGGAGGCAGCAG |
| 16S 806R | GGACTACVVGGGTATCTAATC |

**Supplementary Table 2. List of qPCR primers.**

| Gene name | F primer | R primer |
| --- | --- | --- |
| Human-*JAM2* | TCGTTGTGAAGTTAGTGCCC | CTCTACCACAGTTCCACTCAG |
| Human-*CD206* | GGGTTGCTATCACTCTCTATGC | TTTCTTGTCTGTTGCCGTAGTT |
| Human-*ARG1* | TGGACAGACTAGGAATTGGCA | CCAGTCCGTCAACATCAAAACT |
| Human-*IL10* | TCAAGGCGCATGTGAACTCC | GATGTCAAACTCACTCATGGCT |
| Human-*TGF-β* | CAATTCCTGGCGATACCTCAG | GCACAACTCCGGTGACATCAA |
| Human-*TNF-α* | CCTCTCTCTAATCAGCCCTCTG | GAGGACCTGGGAGTAGATGAG |
| Human-*IL-1β* | TTCGACACATGGGATAACGAGG | TTTTTGCTGTGAGTCCCGGAG |
| Human-*IL-6* | CCTGAACCTTCCAAAGATGGC | TTCACCAGGCAAGTCTCCTCA |
| Human-*CXCL10* | GTGGCATTCAAGGAGTACCTC | TGATGGCCTTCGATTCTGGATT |
| Mouse-*Jam2* | GAGAGTATCGCTGTGAAGTCAG | AGTCATAACAGAAGTGGGCAC |
| Mouse-*Sele* | GCTTCGTGTACCAATGCATC | GCTTCCATAGTCAGGGTGTTC |
| Mouse-*Sema4c* | GAAGAGCAACCAGACCGAATG | ATAGGTGCCGCAGACATACAG |
| Mouse-*Ccl6* | GCTGGCCTCATACAAGAAATGG | GCTTAGGCACCTCTGAACTCTC |
| Mouse-*Hgf* | ACTTCTGCCGGTCCTGTTG | CCCCTGTTCCTGATACACCT |
