# Supplementary material for: Stress accelerates hepatocellular carcinoma progression via a gut microbial-metabolite axis
Source: Front Immunol. 2026 May 12;17:1790214. doi: 10.3389/fimmu.2026.1790214 (PMC13205688; doi:10.3389/fimmu.2026.1790214)
Supplement: Supplementary file 2 [file DataSheet2.docx]

**Supplementary Methods**

**1 Single-Cell RNA Sequencing (scRNA-seq) Analysis**

scRNA-seq was commissioned to Majorbio Bio-Pharm Technology Co. Ltd. (Shanghai, China) and analyzed as previously described(1-10).

**1.1 Library Construction and Sequencing**

Single-cell libraries were generated using the 10x Genomics Chromium system in strict accordance with the manufacturer’s standard protocols. Individual cells were encapsulated into Gel Beads-in-Emulsion (GEMs) containing barcoded beads, which harbored Read1 adapters, 16-base pair (bp) cell barcodes, 12-bp unique molecular identifiers (UMIs), and poly(dT) primers. Following reverse transcription, GEMs were disrupted, and the resulting complementary DNA (cDNA) was purified, amplified, and subjected to library construction and size selection. Library quantity and quality were rigorously assessed using a Qubit 2.0 Fluorometer, Agilent 2100 Bioanalyzer, and quantitative polymerase chain reaction, with a target concentration of 2 nM. Sequencing was performed on an Illumina NovaSeq 6000 platform using 150-bp paired-end (PE150) reads, where Read1 (28 bp) corresponded to barcode/UMI sequences and Read2 (150 bp) captured transcript sequences, with an average sequencing depth of ~150 Gb per sample.

**1.2 Sequencing Quality Control and Preprocessing**

Raw sequencing reads were trimmed to remove adapter sequences, low-quality reads (defined as reads with >20% of bases having a Phred quality score≤5) and reads containing >3 ambiguous bases. Quality assessment of clean reads was conducted using FastQC (v0.11.9).

**1.3 Cell Clustering and Annotation**

Doublet cells were eliminated using Scrublet (v0.2.3) to minimize technical artifacts. Subsequent downstream analyses were performed in R (v4.2.2) utilizing the Seurat package (v4.3.0). Cells with abnormally high mitochondrial gene content (mitochondrial reads > 20%) or low feature counts (detected genes < 200) were excluded to ensure data quality. Gene expression data were normalized using the NormalizeData function (scale.factor = 10,000), and multi-sample integration was implemented using Harmony to mitigate batch effects. Principal component analysis (PCA) was performed for dimensionality reduction, and uniform manifold approximation and projection (UMAP) embedding was generated using the top 10–20 principal components (PCs). Cell clustering was conducted using SCTransform (resolution = 1.0). Marker genes for each cluster were identified via the FindAllMarkers function (average log2 fold change [avg-logFC] > 0.25; adjusted p-value < 0.05) and employed for cell-type annotation based on established lineage-specific markers. Top marker genes were visualized using violin plots, bubble plots, and heatmaps, and their expression distributions across samples were quantified.

**1.4 Differential Expression and Functional Enrichment**

Differential expression analysis was performed using Seurat’s FindMarkers function to compare gene expression profiles. Significant differentially expressed genes (DEGs) were defined as those with adjusted p-value < 0.05 and |log2FC| > 0.15. These DEGs were subjected to Gene Ontology (GO) Biological Process enrichment analysis using the cluster Profiler package. Enrichment analysis was performed using enrichGO with Benjamini–Hochberg correction for multiple testing (adjusted p-value < 0.05, q-value < 0.2).

**1.5 Cell–Cell Communication Analysis**

Cell–cell communication networks were inferred using the CellChat package (v1.6.1) with the Mouse CellChatDB.

**2. Metagenomic sequencing**

A subset of human sample data used in this study has been previously analyzed and reported in another study(11). For human samples, fecal DNA was isolated from frozen stools with the MagMAX™ Microbiome Ultra Nucleic Acid Isolation Kit (Thermo Fisher Scientific, MA, USA). For mice samples, fecal DNA was extracted using Magnetic Soil and Stool DNA Kit (TIANGEN BIOTECH, DP712). DNA purity and integrity verified by agarose gel electrophoresis and concentration were quantified with a NanoDrop 2000. DNA samples of sufficient quality were fragmented to a mean size of ~350 bp. Subsequent library construction was conducted via sequential steps: end repair, A-tailing, adapter ligation, purification, and PCR amplification. Library quality was assessed by quantifying the effective concentration via qPCR. Qualified libraries were sequenced on the Illumina PE150 platform with a target depth was ≥8.0 Gb per sample.

For metagenomic data analyses, adapter trimming and filtering were performed using fastp. Host contaminants were further removed by alignment to the reference genome using Bowtie2 (v2.3.5.1, default parameters; read coverage >80%). Metagenomic microbial composition analyses were performed using the vegan (v2.6-10) R package1. Bray–Curtis distance matrices were constructed to assess differences in microbial community composition among groups, and statistical significance was evaluated using permutational multivariate analysis of variance (PERMANOVA) implemented via the adonis2 function. The Alpha diversity index was calculated using the vegan package. Differential species screening was performed using the microeco (v1.9.1) R package2 based on the Linear Discriminant Analysis Effect Size (LEfSe) method, employing Linear Discriminant Analysis (LDA) to select significant features with LDA > 2 and P < 0.05.

**3 Targeted metabolomics analysis of samples from clinical subjects and mice**

A subset of human sample data used in this study has been previously analyzed and reported in another study(11). Metabolomic profiling of serum samples from clinical participants and mice was conducted by Metabo-Profile Biotechnology (Shanghai, China) using the Q300 Metabolite Array. Standards for all targeted metabolites were sourced from Sigma-Aldrich (St. Louis, MO, USA), Steraloids Inc. (Newport, RI, USA), and TRC Chemicals (Toronto, ON, Canada). For sample pretreatment, 20 μL of serum was vortex-mixed with 120 μL of ice-cold methanol for 5 min to induce protein precipitation. After centrifugation, 30 μL of the supernatant was mixed with 20 μL of freshly prepared derivatization reagents and incubated at 30 °C for 60 min. Post-derivatization, 330 μL of ice-cold 50% (v/v) methanol aqueous solution was added to the mixture, which was then incubated at −20 °C for 20 min. The sample was subsequently centrifuged at 4000 × g for 30 min at 4 °C. A 135 μL aliquot of the resulting supernatant was transferred to a new microplate well and mixed with 10 μL of internal standard solution.

Absolute quantification of metabolites in plasma samples was performed using an ultra-performance liquid chromatography–tandem mass spectrometry (UPLC–MS/MS) system (ACQUITY UPLC-Xevo TQ-S, Waters Corp., Milford, MA, USA). The analytical column was an ACQUITY UPLC BEH C18 column (1.7 μM particle size, 2.1 × 100 mm; Waters). The mobile phase consisted of phase A (water containing 0.1% formic acid, v/v) and phase B (acetonitrile/isopropanol [IPA], 70:30, v/v). The gradient elution program was as follows: 0–1 min, 5% B; 1–11 min, linear gradient from 5% to 78% B; 11–13.5 min, linear gradient from 78% to 95% B; 13.5–14 min, linear gradient from 95% to 100% B; 14–16 min, isocratic elution at 100% B; 16–16.1 min, linear gradient from 100% to 5% B; 16.1–18 min, isocratic re-equilibration at 5% B. The injection volume was 5 μL, flow rate was 0.4 mL/min, column temperature was maintained at 40 °C, and sample manager temperature was set to 40 °C. Electrospray ionization (ESI) was used in both positive (ESI+) and negative (ESI−) modes. Source temperature was 150 °C, desolvation temperature was 550 °C, and desolvation gas flow rate was 1000 L/h.

Raw UPLC–MS/MS datasets were processed with MassLynx software (v4.1, Waters Corp., Milford, MA, USA) to perform peak integration, calibration curve generation, and absolute quantification of individual metabolites. A total of 203 metabolites were reliably detected in samples from clinical participants, whereas 204 reliably detected metabolites were identified in mouse samples. Both datasets were subjected to subsequent analyses.

To quantify the overall abundance of tryptophan pathway-related metabolites, a tryptophan pathway score was calculated for each sample based on the seven measured tryptophan-related metabolites in our dataset. Briefly, the abundance of each metabolite was first standardized across all samples using Z-score transformation according to the following formula:

$$Z_{ij}=\frac{x_{ij}-\mu_{j}}{\sigma_{j}}$$

where $x_{ij}$represents the abundance of metabolite $j$in sample $i$, and $\mu_{j}$and $\sigma_{j}$denote the mean and standard deviation of metabolite $j$across all samples, respectively.

The tryptophan pathway score for each sample was then defined as the average of the Z-scored values of the seven metabolites:

$$\text{Tryptophan pathway score}_{i}=\frac{1}{7}\sum_{j=1}^{7} Z_{ij}$$

**4 Behavioral tests**

**4.1 SPT**

The sucrose preference test (SPT) was used to assess the degree of pleasure loss in mice as previously described(12). Briefly, during adaptation, mice in their home cages are given 48 h of continuous exposure to two regular bottles, one containing sucrose water (1% (wt/vol)) and one containing tap water (regular water). All mice also have ad libitum access to lab chow. After this, mice are transferred to the chambers of the SPT apparatus for apparatus adaptation for 24 h. During the baseline measurement stage, mice are moved into a temperature-controlled room that should be used only for the test and are transferred to the SPT apparatus. Each mouse is given one tube of sucrose water (1% (wt/vol)) and one tube of regular water for 12 h. The baseline measurement stage lasts for 48 hours. During the preference test after performing baseline measurements, mice are deprived of both food and water for 12 h. Immediately after deprivation, all animals are given 12-h access to one tube of 1% (wt/vol) sucrose solution and one tube of regular water. Each tube is weighed before and after the test. At the end of the test, all animals are returned to group housing with ad libitum food and water. Because of the effect of circadian rhythms on the drinking of mice, all measurements of SPT were performed at night. Sucrose water preference (%) = sucrose water consumption/ (sucrose water consumption + distilled water consumption) x 100%.

**4.2 FST**

The forced swimming test (FST) was used to assess the degree of desperation in mice, as previously described(13). Mice were carefully introduced one at a time into a cylindrical water tank (30 cm high x 15 cm diameter, water temperature 23 ± 1 ℃). The mice were allowed to remain in the water tank for 6 min, and the immobility time was recorded for the last 4 min. Immobility time was defined as when the body was perpendicular to the water surface with only the nose sticking out or when the mice floated and moved slightly on the water's surface. Scoring of immobility time was performed by means of automated video tracking software (EthoVision XT8, Noldus, Netherlands) as described previously.

**4.3 TST**

The tail suspension test (TST) was performed to evaluate depression-like behavior. The mice were suspended for a period of 6 min, and the time spent immobile during the last 4 min of 6 min period was recorded. Scoring of immobility time was performed by means of automated video tracking software (EthoVision XT8, Noldus, Netherlands) as described previously(13).

**5 Abx**

Antibiotic cocktails in drinking water (containing 100 µg/ml neomycin, 50 µg/ml streptomycin, 100 µg/ml ampicillin, 50 µg/ml vancomycin, 100 µg/ml metronidazole, 1 mg/ml bacitracin, 125 µg/ml ciprofloxacin, and 100 µg/ml ceftazidime) were freshly prepared every two days and were given ad libitum for 10 days to C57BL/6J mice. This protocol was reported that it would not promote loss of body weight(14).

**6 Fecal microbiota transplantation (FMT) from human**

Fresh stool samples were collected from individuals with MDD or health control, as described previously(15). Briefly, stool (20 mg) was dissolved in 1 mL saline, vigorously mixed for 3 min, centrifuged for 3 min at 4℃, and 200 μL supernatant collected. 10 % volume of glycerol was added to the fecal suspension, and it was sub-packaged and frozen at -80 ℃. Mice were administered 200 μL of the above supernatant by gavage every other day for the next 14 days after 3d of antibiotics treatment.

**7 Fecal microbiota transplantation from mice**

Following an established protocol with minor modifications(16), fresh feces from stress group (n=10) and con group (n=10) mice were collected and resuspended in sterile PBS solution at a ratio of 40 mg/mL. The solution was vigorously mixed for 3 min before centrifugation at 500×g for 3 min. Subsequently, the supernatant was collected and filtered through a 70 mm cell strainer. 10 % volume of glycerol was added to the fecal suspension, and it was sub-packaged and frozen at -80 ℃. After 3d of antibiotics treatment, each Abx-treated mouse was given 200 μl of fecal suspension by oral gavage every other day for the next 14 days.

**8 Isolation of mouse liver ECs**

As described previously, mice were sacrificed and the livers were minced and dissociated with HBSS (Gibco) containing dispaseⅡ (Sigma), collagenaseⅠ(Sigma), collagenaseⅡ(Sigma) and DNaseⅠ(Sigma) at 37°C for 20 min at indicated time points(17, 18). To remove connective tissue, the resulting cell suspension was filtered through a 70 μm filter to obtain single cell suspension and cells were collected at 400 g for 5 min. After the cells were resuspended in ammonium-chloride-potassium (ACK) lysis buffer for 5 minutes on ice, hepatocytes and NPCs were then separated by an additional centrifugation step at 50g for 5 min at 4 °C. NPCs in the supernatant were utilized for flow cytometry analysis and bead isolation. The total NPCs were centrifuged and resuspended with MACS buffer. The obtained single cell suspension was incubated with Dynabeads magnetic beads (Invitrogen) coated with rat anti-mouse CD31 antibodies (BD Bioscience). After all treatments, the CD31+ cells (ECs) were collected and subjected to qPCR analysis(19, 20).

**9 Immunostaining**

Mouse tumor tissues were embedded in OCT compound and cryopreserved for histological analysis. Tumor tissues were sectioned at 8 μm slides, and the slides were blocked with 5% donkey serum followed by incubation with an anti-VE-cadherin polyclonal primary antibody (#AF1002, R&D Systems) at 4°C overnight. After incubation with fluorophore-conjugated secondary antibodies (#705-605-147, Jackson ImmunoResearch), nuclei were counterstained with DAPI (#10236276001, Roche), and the sections were mounted with an antifade mounting medium. Images were acquired using an Axiovert LSM980 confocal microscope (Zeiss).

**10 Flow cytometry**

The NPCs were incubated with the following conjugated antibodies: FITC Rat Anti-Mouse CD31 (#553372, BD), PerCP-Cy™5.5 Anti-Mouse CD45 (#550994, BD), PE Rat anti-mouse F4/80 (#565410, BD), PE-Cyanine7 Anti-Mouse CD11b(#25-0112-82, eBioscience) in the 4℃ refrigerator for 30 min and washed twice with cold PBS (1% FBS). The incubated cells were then analyzed using flow cytometry on the BD LSRFortessa. The obtained data were processed using Flow Jo-V10 (BD Bioscience).

**11 Cell culture and lentiviral transduction**

HUVECs were cultured in EC specific medium (Milipore, SCME-BM). To perform gene overexpression in HUVECs, the open reading frame of the human JAM2 gene was subcloned into the lentiviral vector as previously described(21, 22). A negative control vector was also constructed using a scrambled sequence provide by Sigma, which does not match any genomic sequence. Lentiviral particles were generated by co-transfecting 2.5 mg of total DNA, including the shuttle lentiviral vector loaded with the target gene overexpression or scrambled sequence, PSPA.2 and PMP2.D, into 293T cells using liposome transfection. Viral supernatants were collected and used to transduce HUVECs.

**12 Co-immunoprecipitation (Co-IP) Assay**

Plasmids were transfected into HEK293T cells using Lipo6000 (PEI; Polysciences). GFP-empty vector served as negative control for non-specific interactors. Forty-eight hours post-transfection, cells were washed twice with ice-cold PBS and lysed at 4 °C for 20 min in IP buffer (1% (v/v) Triton X-100, 20 mM HEPES pH 7.4, 140 mM KCl, 5 mM MgCl₂ and EDTA-free protease inhibitor; 600 µL per 10 cm dish). Lysates were cleared by centrifugation, 50 µL aliquot of supernatant was retained as input; the remainder was incubated overnight at 4 °C with GFP-Trap magnetic agarose (ChromoTek) generated by pre-conjugating GFP antibody to Protein A 4FF Sefinose™ Resin (Sangon Biotech, C600957). Beads were washed three times with IP buffer and bound proteins eluted in 4× Laemmli sample buffer at 95 °C for 5 min. For immunoblot analysis, 20% of the eluate was used to detect GFP, and the remaining 80% was used to detect co-immunoprecipitated proteins.

**13 Co-culture of HUVECs and THP-1 monocytes**

Co-culture of HUVECs and THP-1 monocytes were performed as previously described(23, 24). Briefly, the THP-1 monocytes (ATCC) were seeded onto cell culture inserts with a 0.4 mm pore size PC membrane (Beyotime, China) and differentiated into macrophages by the treatment with phorbol 12-myristate 13-acetate (PMA; Sigma, USA) for 24 h as previously described. The HUVECs were seeded onto 12-well plates. After adhesion of the HUVECs, the differentiated THP-1 monocytes seeded on the cell culture inserts were transferred to the 12-well plates to initiate co-culturing with the HUVECs. Then, the HUVECs were incubated with 500 μmol/L IPA. The co-culture was incubated in a CO2 incubator for 2 days, the supernatants from the upper chamber of co-culture were collected for ELISA analysis and cells were collected for RNA extraction and qPCR analysis. As for the co-culture of JAM2-overexpressing HUVECs with the differentiated THP-1 monocytes, JAM2 overexpression viral supernatants were transduced into HUVECs before the co-culturing.

**14 Total RNA extraction and qPCR with reverse transcription**

Isolated Liver ECs and cell pellets were collected for RNA extraction. Total RNA was prepared using TRIzol (Invitrogen) and converted to cDNA using PrimeScript RT reagent Kit (Takara, RR047A-1). qPCR was conducted using SYBR green on the CFX96 Real-time PCR Detection System (Bio-Rad) as previously described.

**15 ELISA**

To quantify IL-10 and TGF-β levels, the supernatants from the upper chamber of co-culture were collected and used for ELISA with IL-10 and TGF-β human pre-coated ELISA kits (ThermoFisher), following the manufacturer’s instructions, and absorbance was read at 450 nm. The concentration of IL-10 and TGF-β in supernatants was determined from a standard curve generated by serial dilution of IL-10 or TGF-β, respectively.

References

1. Zheng C, Zheng L, Yoo J-K, Guo H, Zhang Y, Guo X, et al. Landscape of Infiltrating T Cells in Liver Cancer Revealed by Single-Cell Sequencing. Cell. 2017;169(7):1342–56.e16.

2. Zhang Q, He Y, Luo N, Patel SJ, Han Y, Gao R, et al. Landscape and Dynamics of Single Immune Cells in Hepatocellular Carcinoma. Cell. 2019;179(4):829–45.e20.

3. Cappuyns S, Piqué-Gili M, Esteban-Fabró R, Philips G, Balaseviciute U, Pinyol R, et al. Single-cell RNA sequencing-derived signatures define response patterns to atezolizumab + bevacizumab in advanced hepatocellular carcinoma. Journal of Hepatology. 2025;82(6):1036–49.

4. Hao X, Zheng Z, Liu H, Zhang Y, Kang J, Kong X, et al. Inhibition of APOC1 promotes the transformation of M2 into M1 macrophages via the ferroptosis pathway and enhances anti-PD1 immunotherapy in hepatocellular carcinoma based on single-cell RNA sequencing. Redox Biology. 2022;56.

5. Brown J, Pirrung M, McCue LA, Wren J. FQC Dashboard: integrates FastQC results into a web-based, interactive, and extensible FASTQ quality control tool. Bioinformatics. 2017;33(19):3137–9.

6. Wu T, Hu E, Xu S, Chen M, Guo P, Dai Z, et al. clusterProfiler 4.0: A universal enrichment tool for interpreting omics data. The Innovation. 2021;2(3).

7. Butler A, Hoffman P, Smibert P, Papalexi E, Satija R. Integrating single-cell transcriptomic data across different conditions, technologies, and species. Nature Biotechnology. 2018;36(5):411–20.

8. Tan Z, Chen X, Zuo J, Fu S, Wang H, Wang J. Comprehensive analysis of scRNA-Seq and bulk RNA-Seq reveals dynamic changes in the tumor immune microenvironment of bladder cancer and establishes a prognostic model. Journal of Translational Medicine. 2023;21(1).

9. Jin S, Guerrero-Juarez CF, Zhang L, Chang I, Ramos R, Kuan C-H, et al. Inference and analysis of cell-cell communication using CellChat. Nature Communications. 2021;12(1).

10. Jin S, Plikus MV, Nie Q. CellChat for systematic analysis of cell–cell communication from single-cell transcriptomics. Nature Protocols. 2024;20(1):180–219.

11. Du J-Y, Zhang Z-J, Tan L, Yang J-Y, Yang R-N, Chen Y-L, et al. Gut microbiota dysbiosis and metabolic perturbations of bile/glyceric acids in major depressive disorder with IBS comorbidity. mBio. 2025;16(11):e02447–25.

12. Liu M-Y, Yin C-Y, Zhu L-J, Zhu X-H, Xu C, Luo C-X, et al. Sucrose preference test for measurement of stress-induced anhedonia in mice. Nature Protocols. 2018;13(7):1686–98.

13. Tomida S, Mamiya T, Sakamaki H, Miura M, Aosaki T, Masuda M, et al. Usp46 is a quantitative trait gene regulating mouse immobile behavior in the tail suspension and forced swimming tests. Nature Genetics. 2009;41(6):688–95.

14. Koh A, Molinaro A, Ståhlman M, Khan MT, Schmidt C, Mannerås-Holm L, et al. Microbially Produced Imidazole Propionate Impairs Insulin Signaling through mTORC1. Cell. 2018;175(4):947–61.e17.

15. Qi X, Yun C, Sun L, Xia J, Wu Q, Wang Y, et al. Gut microbiota–bile acid–interleukin-22 axis orchestrates polycystic ovary syndrome. Nature Medicine. 2019;25(8):1225–33.

16. Bárcena C, Valdés-Mas R, Mayoral P, Garabaya C, Durand S, Rodríguez F, et al. Healthspan and lifespan extension by fecal microbiota transplantation into progeroid mice. Nature Medicine. 2019;25(8):1234–42.

17. Naito H, Wakabayashi T, Ishida M, Gil C-H, Iba T, Rahmawati FN, et al. Isolation of tissue-resident vascular endothelial stem cells from mouse liver. Nature Protocols. 2020;15(3):1066–81.

18. Qing J, Ren Y, Zhang Y, Yan M, Zhang H, Wu D, et al. Dopamine receptor D2 antagonism normalizes profibrotic macrophage-endothelial crosstalk in non-alcoholic steatohepatitis. Journal of Hepatology. 2022;76(2):394–406.

19. Mo C, Li H, Yan M, Xu S, Wu J, Li J, et al. Dopaminylation of endothelial TPI1 suppresses ferroptotic angiocrine signals to promote lung regeneration over fibrosis. Cell Metabolism. 2024;36(8):1839–57.e12.

20. Chen Y, Pu Q, Ma Y, Zhang H, Ye T, Zhao C, et al. Aging Reprograms the Hematopoietic-Vascular Niche to Impede Regeneration and Promote Fibrosis. Cell Metabolism. 2021;33(2):395–410.e4.

21. Ding B-S, Nolan DJ, Butler JM, James D, Babazadeh AO, Rosenwaks Z, et al. Inductive angiocrine signals from sinusoidal endothelium are required for liver regeneration. Nature. 2010;468(7321):310–5.

22. Ding B-S, Cao Z, Lis R, Nolan DJ, Guo P, Simons M, et al. Divergent angiocrine signals from vascular niche balance liver regeneration and fibrosis. Nature. 2013;505(7481):97–102.

23. Zhou X, Zhang C, Yang S, Yang L, Luo W, Zhang W, et al. Macrophage-derived MMP12 promotes fibrosis through sustained damage to endothelial cells. Journal of Hazardous Materials. 2024;461.

24. Chen G, Shen Y, Li X, Jiang Q, Cheng S, Gu Y, et al. The endoplasmic reticulum stress inducer thapsigargin enhances the toxicity of ZnO nanoparticles to macrophages and macrophage-endothelial co-culture. Environmental Toxicology and Pharmacology. 2017;50:103–10.
